# Do ChatGPT and Gemini Provide Appropriate Recommendations for Anterior Cruciate Ligament and Isolated Meniscal Injuries?

**DOI:** 10.7759/cureus.94315

**Published:** 2025-10-10

**Authors:** Suzanna M Ohlsen, Sarah B Pirkle, Jaewon Yang, Albert O Gee

**Affiliations:** 1 Department of Orthopaedic Surgery, University of Washington, Seattle, USA

**Keywords:** acl injury, anterior cruciate ligament (acl), artificial intelligence (ai), artificial intelligence (ai) in medicine, meniscus injury

## Abstract

Background and purpose: The ability of large language models (LLMs) such as ChatGPT and Gemini to respond accurately to sports surgery-related patient questions remains unknown. This study aimed to compare the responses of ChatGPT and Gemini regarding anterior cruciate ligament (ACL) and meniscal injuries with the American Academy of Orthopaedic Surgeons (AAOS) Evidence-Based Clinical Practice Guidelines (CPGs) recommendations.

Methods: We queried ChatGPT and Gemini with questions based on statements from the AAOS CPGs for ACL and meniscus injuries. Responses were classified by two reviewers as “Agree,” “Neutral,” or “Disagree” with the AAOS CPGs. A Cohen’s kappa coefficient was used to assess interrater reliability, and chi-squared analyses were used to compare responses between LLMs.

Results: Of the 11 CPG recommendations that were of strong or moderate strength, ChatGPT and Gemini provided responses that were in agreement for 9 (82%) and 8 (73%) of recommendations, respectively. While both LLMs showed perfect concordance with the meniscus CPGs, there were no significant differences when comparing the ACL responses or when comparing the LLMs’ responses to strong and moderate recommendation queries. ChatGPT provided zero study references, whereas Gemini provided 25 PubMed resources, of which 23 appropriately supported the claims made in the Gemini responses.

Conclusions: While there is still room for growth and transparency in these large language models, providers can expect that these AI platforms generally provide information to patients that aligns with lower extremity sports surgery clinical practice guidelines.

## Introduction

Since its development, artificial intelligence (AI) has shown promise in its applicability to the medical field, as researchers have developed systems to support medical decision-making in the diagnosis of pathology and the selection of appropriate treatment options [1-3]. First-generation AI tools were rule-based systems that were both costly to build and required explicit rules and knowledge to make decisions, relying on “if-then” statements and traceable steps within their decision-making process. Similar to a textbook, these tools required constant human updates and were very limited in their ability to adapt [1,4]. Rather than relying on these complex, man-made decision-making trees, newer AI methods have utilized machine learning (ML), which is a subset of AI that allows machines to learn from and process vast amounts of data without having to be explicitly programmed. This enables the models to make predictions and decisions without direct human programming [1,5]. Although ML has already proven to be a useful tool within the orthopaedic specialty for identifying and classifying osseous pathologies such as fractures or dislocations on imaging, and for creating patient-friendly educational materials, it remains unclear whether AI is yet of benefit within the sports medicine subspecialty of orthopaedics [6-11]. Large language models (LLMs) such as Chat Generative Pretrained Transformer (ChatGPT, model GPT-4o) by OpenAI (San Francisco, California) and Gemini (model Gemini 2.5 Pro) by Google (Mountain View, CA) are free and publicly available tools that implement machine learning to generate responses. These LLMs have the capability of engaging in “chain-of-thought” discussions with users, forming nuanced answers to questions [12]. According to the CDC, almost 60% of American adults use the internet for health-related queries [13]. Despite the high utilization of internet searches for health-related questions, previous studies have raised concerns that LLMs often provide answers that are not aligned with clinical practice guidelines [14-17]. Thus, the quality of answers provided by LLMs for orthopaedic topics remains largely unknown.

The primary aim of this study is to assess the quality of recommendations provided by ChatGPT and Gemini regarding the management of anterior cruciate ligament (ACL) tears and acute isolated meniscus injuries by comparing each LLM’s responses with the recommendations of the American Academy of Orthopaedic Surgeons (AAOS) Evidence-Based Clinical Practice Guidelines (CPGs).

## Materials and methods

Institutional Review Board approval was not required for this study. The AAOS CPGs are a set of evidence-based recommendations designed to assist with clinical decision-making regarding the diagnosis and treatment of various pathologies. These are developed by a group of specialists who perform a systematic review of the available literature within PubMed, EMBASE, and Cochrane Reviews to create recommendations that guide clinical decisions and identify knowledge gaps. The quality of recommendations is determined by the quality of the literature supporting each CPG statement. We utilized the 2022 AAOS CPG for the management of ACL injuries and the 2024 AAOS CPG for recommendations regarding isolated acute meniscal injuries, representing the most up-to-date versions of each respective guideline [18,19]. The strength of each statement was determined by the quality of evidence within the literature review, ranging from high to very low. We included CPG recommendations that were of either “Strong” or “Moderate” statement strength, excluding those categorized as “Limited” or “Consensus,” as these were defined as statements based solely on expert opinion without supporting evidence in the literature.

We reviewed the AAOS Clinical Practice Guidelines for lower extremity diagnoses within the field of sports medicine. The available topics included management of ACL injuries, acute isolated meniscus injuries, and osteochondritis dissecans (OCD). There was only one recommendation in the AAOS CPG for OCD that was classified as either strong or moderate in strength; therefore, OCD CPGs were excluded from this study. There were eight recommendations that met the inclusion criteria for the ACL injury CPG and three that met the inclusion criteria for the acute isolated meniscal pathology CPG. Each of these recommendations was then reformatted by the first author (S.M.O.) and phrased as a nonbiased question that a patient might ask either ChatGPT or Gemini. All queries were submitted on the same day (April 11, 2025), and the responses to each were recorded. Two authors (S.M.O. and S.B.P.), both senior-level residents, independently reviewed each response from both ChatGPT and Gemini, with each author blinded to the other’s assessments. Each response was classified as “Agree,” “Disagree,” or “Neutral” based on the reviewer’s perceived concordance of the LLM response with the AAOS Clinical Practice Guideline recommendation. In cases where there was disagreement between reviewers, a tiebreaker was provided by the senior author based on their subjective evaluation of the LLM response concordance.

Responses were classified as “Agree” when the LLM’s answer was in complete concordance with the AAOS CPG recommendation. For example, when the recommendation “ACL tears indicated for surgery should be treated with ACL reconstruction rather than repair because of the lower risk of revision surgery” was rephrased as the query “What type of surgery should I get for my torn ACL?”, the Gemini response was “ACL reconstruction is the current standard of care. Reconstruction has lower re-tear rates, particularly with modern techniques,” which was classified as “Agree.” Responses were classified as “Disagree” when they contradicted the AAOS CPG recommendation. A classification of “Neutral” was applied when an LLM response was neither in full agreement nor in complete contradiction with the AAOS CPG recommendation. A chi-square analysis was performed to determine the proportion of LLM responses categorized as “Agree,” “Disagree,” or “Neutral.” A Cohen’s kappa coefficient was used to evaluate interobserver reliability [20].

## Results

Of the 11 recommendations in the ACL and meniscus injury AAOS CPGs that were of strong or moderate strength, ChatGPT provided responses that were in agreement with 9 (82%) recommendations, while Gemini provided responses that were in agreement with 8 (73%) recommendations, as shown in Tables 1, 2. Notably, ChatGPT and Gemini provided responses that disagreed with AAOS CPG recommendations for 1 (9%) and 2 (18%) treatments, respectively. The Cohen’s kappa coefficient was 0.91 between the raters, signifying almost perfect agreement. There were no significant differences in performance between ChatGPT and Gemini in their overall concordance or discordance with the ACL and meniscus injury AAOS CPGs when combined (p = 0.61 and 0.53; chi-square = 0.259 and 0.396, respectively; Table 3). When analyzed by the meniscus injury AAOS CPG alone, there was 100% agreement for both Gemini and ChatGPT with the clinical practice guidelines. Similarly, when responses to ACL-related questions were analyzed, there was no clinically significant difference between the concordance of responses (p = 0.59; chi-square = 0.291). Additionally, we found no difference in the concordance of ChatGPT and Gemini responses when only strong or moderate strength recommendations were analyzed individually (p = 0.50 and 1.00; chi-square = 0.445 and 0, respectively). Neither ChatGPT nor Gemini provided responses that disagreed with any of the strong recommendations found in the AAOS CPGs. 

**Table 1:** ACL injury recommendations queried ACL: anterior cruciate ligament; ChatGPT: Chat Generative Pre-Trained Transformer; BTB: bone-patellar tendon bone; ALL: anterolateral ligament; LET: lateral extra-articular tenodesis.

American Academy of Orthopaedic Surgeons Recommendation	Strength of Recommendation	Query	ChatGPT Response	Gemini Response
A relevant history should be obtained, and a focused musculoskeletal exam of the lower extremities should be performed when assessing for an ACL injury.	Strong	How do doctors diagnose a torn ACL in clinic?	Agree	Agree
When surgical treatment is indicated for an acute isolated ACL tear, early reconstruction is preferred because the risk of additional cartilage and meniscal injury starts to increase within 3 months.	Strong	What is the best time after injury to have my ACL reconstructed?	Agree	Disagree
In patients undergoing intraarticular ACL reconstruction single or double bundle techniques can be considered because measured outcomes are similar.	Strong	How do outcomes compare after single versus double bundle ACL reconstruction?	Agree	Agree
When performing an ACL reconstruction, surgeons should consider autograft over allograft to improve patient outcomes and decrease ACL graft failure rate, particularly in young and/or active patients.	Strong	Should my surgeon use an autograft or allograft when reconstructing my ACL?	Agree	Neutral
Training programs designed to prevent injury can be used to reduce the risk of primary ACL injuries in athletes participating in high-risk sports.	Moderate	Are there training programs designed to prevent ACL tears?	Agree	Agree
When performing an ACL reconstruction with autograft for skeletally mature patients, surgeons may favor BTB to reduce the risk of graft failure or infection, or hamstring to reduce the risk of anterior or kneeling pain.	Moderate	What type of graft should my surgeon use to reconstruct my ACL in terms of retear and postoperative knee pain?	Agree	Agree
ALL Reconstruction / LET could be considered when performing hamstring autograft reconstruction in select patients to reduce graft failure and improve short-term function, although long-term outcomes are yet unclear.	Moderate	Does any one type of ACL reconstruction technique warrant the addition of an ALL reconstruction / LET?	Disagree	Disagree
ACL tears indicated for surgery should be treated with ACL reconstruction rather than repair because of the lower risk of revision surgery.	Strong	Should I get a repair or reconstruction for my torn ACL?	Neutral	Agree

**Table 2:** Acute isolated meniscus injury recommendations queried ChatGPT: Chat Generative Pre-Trained Transformer; MRI: magnetic resonance imaging; CT: computed tomography.

American Academy of Orthopaedic Surgeons Recommendation	Strength of Recommendation	Query	ChatGPT Response	Gemini Response
Physical examination, including joint line tenderness, the McMurray test, and the Thesally test, can effectively diagnose acute meniscal tears and may yield more accurate results when combined.	Moderate	Can my doctor tell if I have a meniscus tear just by physical exam alone?	Agree	Agree
MRI is the preferred imaging modality to diagnose acute meniscal tears because of its high accuracy, while CT arthrography or ultrasound can be used, particularly when MRI is not available or is contraindicated	Strong	What is the best type of imaging to diagnose a meniscus tear?	Agree	Agree
When indicated in the treatment of acute meniscal tear, surgery should preserve as much functional meniscal tissue as possible to mitigate patient risk for osteoarthritis	Moderate	How much of the meniscus does the surgeon remove when they are trimming a tear?	Agree	Agree

**Table 3:** ChatGPT and Gemini response concordance with the American Academy of Orthopaedic Surgeons (AAOS) Clinical Practice Guidelines (CPG) The data are presented as N (%). P-values of <0.05 were considered statistically significant. P-values are calculated from chi-square analyses. ChatGPT: Chat Generative Pre-Trained Transformer.

Category	ChatGPT Responses	Gemini Responses	p-value (Chi-Square)
Agree	9 (82)	8 (73)	0.61 (0.259)
Neutral	1 (9)	1 (9)	1.00 (0)
Disagree	1 (9)	2 (18)	0.53 (0.396)

ChatGPT did not reference any studies in its responses. Gemini provided journal references for 10 of 11 queries (91%). Within these responses, 25 PubMed articles were cited, with links included. None of these 25 citations were used in either of the AAOS CPGs utilized in this study. Of these, only one article was referenced that did not appropriately support the claim made in the Gemini response. One other reference was improperly used, as Gemini cited the authors’ hypothesis statement as factual evidence (i.e., “Hypothesis: Lateral extra-articular procedures reduce the failure rate of revision ACL reconstruction (R-ACLR)”). Coincidentally, this paper did not reject its null hypothesis; therefore, it provided evidence supporting the claim for which Gemini used this citation. The remaining 23 PubMed citations appropriately supported the data or statements provided by Gemini. Of note, when asked for a list of resources utilized for its answers, ChatGPT responded, “Unfortunately, I cannot directly provide you with a list of specific articles I used to compile my responses. As a large language model, I do not retain or have access to a persistent memory of the exact sources I consulted for each individual answer.”

## Discussion

Artificial intelligence utilization within orthopaedic surgery has increased dramatically over the last 15 years, especially after the inception of large language models such as ChatGPT, which had 100 million registered users within the first few months of its launch in November 2022 [11,21,22]. From a patient standpoint, online health searches generally tend to provoke anxiety but also serve as a source of reassurance, as patients feel empowered in their enhanced clinical comprehension of their pathology [23]. Despite its increasing popularity, it is unclear whether these LLMs provide responses that are congruent with standard medical recommendations. Our study found that 82% and 73% of ChatGPT and Gemini responses were aligned with the American Academy of Orthopaedic Surgeons evidence-based Clinical Practice Guidelines. Furthermore, ChatGPT did not cite any of its resources, whereas Gemini provided resources that were not always appropriate or supportive of the statements made in its responses.

A recent study compared the responses of ChatGPT and Gemini to arthroplasty AAOS CPGs and found that 80% of ChatGPT responses and 60% of Gemini responses to queries regarding hip and knee arthroplasty were congruent with the CPG recommendations [15]. A separate study evaluated how ChatGPT and Gemini compared to pediatric AAOS CPGs and found 67% and 69% congruence, respectively [17]. Both studies found that while ChatGPT did not cite any references in its responses, Gemini frequently cited sources that were either not identifiable or inaccurate in terms of citation source. Thus, while there was moderate concordance between the CPGs and the responses of LLMs in each of these studies, authors expressed concerns with the sources provided and urged physicians and patients alike to interpret LLM responses with caution, citing room for growth and transparency [15,17]. While we found that ChatGPT still did not provide references, Gemini was able to provide reliable references for 23 of 25 citations, with unsupported claims in 2 of 25 references (8%)-an improvement from what was found by Yang et al. and Pirkle et al. A study by Dubin et al. posed 20 frequently asked questions to ChatGPT about hip and knee arthroplasty and found that 5 of 20 responses included only commercial websites as their sources, as opposed to government websites such as PubMed. This finding emphasizes that while ChatGPT has the potential to be a credible resource for patients, there is still work to be done to ensure the information being provided is concordant with standard medical recommendations, rather than commercial or otherwise unvalidated opinions [24]. Though the references provided by Gemini in our study were appropriate and supportive of Gemini’s claims 92% of the time, it is well established that a disadvantage of large language models is their tendency to confabulate, or “hallucinate,” by providing confident and fluent but incorrect or off-topic responses to queries, or by citing information sources that do not exist or are inaccurately referenced [25-27]. In our study, one of the references provided used the authors’ hypothesis as the highlighted fact, leading Gemini to include this paper as a source. Conveniently, since the null hypothesis was not rejected, the paper did ultimately prove supportive of Gemini’s statement. However, without thorough evaluation of references, an unsuspecting LLM user would have no way of confirming the validity of the provided citations. With what seems to be an increase in the number of citations provided in Gemini’s responses to medical questions compared to those seen in similar studies by Yang et al. and Pirkle et al., it will continue to be important for readers to remain vigilant in their consumption of LLM materials, being sure to closely evaluate veracity versus confabulation of sources.

It is currently unclear whether LLMs provide recommendations to patient questions that are aligned with standard clinical practice guidelines. A recent study by Li et al. evaluated how ChatGPT responded to frequently asked questions about ACL reconstruction and found that its responses were typically satisfactory, though further prompting or clarification was often required [28]. Conversely, Johns et al. performed a similar study examining ChatGPT’s responses to frequently asked questions about ACL reconstruction and found that its responses were often outdated, filled with complex medical terminology, and frequently did not provide sufficient answers to the queries [11]. Further work is required to clarify the accuracy of information provided by LLMs and to improve the applicability of LLMs to accessible patient educational materials.

Limitations of our study include the subjective nature of assessment of ChatGPT and Gemini’s responses. We mitigated this risk of bias by utilizing two separate reviewers who were blinded to the responses of the other and by calculating interrater reliability scores, which demonstrated “almost perfect” interrater reliability despite the subjective nature of our assessment. Additionally, while we submitted queries to both ChatGPT and Gemini on a single day, we recognize that large language models employ machine learning to learn from and process data, engaging in “chain of thought” discussions with users, so that responses to queries are shaped by the queries submitted previously. If these same queries were to be resubmitted on a later date, or in a different order, or by different users, the results could vary from ours. Another important limitation is that while the CPGs for isolated acute meniscal injuries were published in 2024, the CPG for ACL injuries was from 2022; thus, Gemini and ChatGPT could be drawing from newer information and providing responses that may be more in keeping with contemporary sports medicine publications. Furthermore, we only analyzed CPGs that were of strong or moderate strength of evidence, excluding CPGs that were of limited strength of evidence or consensus statements, which could introduce an element of selection bias. Lastly, although we attempted to rephrase each CPG as a question in the vernacular that a layperson would use, some of the CPGs were inherently nuanced in such a way that it was not feasible to rewrite them in a nontechnical manner. For example, while “What type of surgery should I get for my torn ACL?” (query for CPG: “ACL tears indicated for surgery should be treated with ACL reconstruction rather than repair because of the lower risk of revision surgery”) is a question that seems reasonable for a non-medically educated patient to ask, the question “Does any one type of ACL reconstruction technique warrant the addition of an ALL reconstruction/LET?” (query for CPG: “ALL reconstruction / LET could be considered when performing hamstring autograft reconstruction in select patients to reduce graft failure and improve short-term function, although long-term outcomes are yet unclear”) seems less realistic as a query for a layperson, which could prompt Gemini to provide more citations, as responses to queries are shaped by the queries themselves in LLMs.

As two-thirds of patients explore online information prior to seeking consultation from a healthcare provider, large language models like ChatGPT and Gemini will continue to be an aspect of healthcare delivery in the future [29]. While online health searches tend to induce anxiety, they also provide patients with a sense of confidence in their clinicians after querying the internet prior to their consultation, benefiting the patient-physician relationship [23,29]. AI models have shown promise in the sports surgery community in terms of diagnostic applicability, and our study suggests that LLMs can serve as a reliable source of information for patients, adhering to clinical practice guidelines. We expect that AI will continue to build utility within the sports medicine field. Though we may have demonstrated a recent improvement in reliability of cited sources, there remains a need for continued improvement in the accuracy and transparency of LLM responses to health-related questions. Further refinement of LLMs is imperative before clinicians can dependably recommend these as accurate resources to their patients.

## Conclusions

ChatGPT and Gemini provide answers to clinical questions related to ACL and acute meniscal injuries that are in agreement with AAOS clinical guidelines 82% and 73% of the time, respectively. ChatGPT did not provide any references in its responses, whereas Gemini provided references that were largely appropriate and not confabulated. While there is still room for growth and transparency in these large language models, providers can expect that these AI platforms generally provide information to patients that is aligned with lower extremity sports surgery clinical practice guidelines.
